# Application of Deep Learning-Based Multimodal Data Fusion for the Diagnosis of Skin Neglected Tropical Diseases: Systematic Review

**DOI:** 10.2196/67584

**Published:** 2025-12-04

**Authors:** G Yohannes Minyilu, Mohammed Abebe Yimer, Million Meshesha

**Affiliations:** 1 Faculty of Computing & Software Engineering Arba Mich Institute of Technology Arba Minch University Arba Minch Ethiopia; 2 Department of Information Science, School of Information Science Addis Ababa University Addis Ababa Ethiopia

**Keywords:** skin NTDs, deep learning–based diagnostics, multimodal data fusion, CNNs, transformer-based models, resource-constrained areas

## Abstract

**Background:**

Neglected tropical diseases (NTDs) are the most prevalent diseases and comprise 21 different conditions. One-half of these conditions have skin manifestations, known as skin NTDs. The diagnosis of skin NTDs incorporates visual examination of patients, and deep learning (DL)–based diagnostic tools can be used to assist the diagnostic procedures. The use of advanced DL-based methods, including multimodal data fusion (MMDF) functionality, could be a potential approach to enhance the diagnostic procedures of these diseases. However, little has been done toward the application of such tools, as confirmed by the very few studies currently available that implemented MMDF for skin NTDs.

**Objective:**

This article presents a systematic review regarding the use of DL-based MMDF methods for the diagnosis of skin NTDs and related diseases (non-NTD skin diseases), including the ethical risks and potential risk of bias.

**Methods:**

The review was conducted based on the PRISMA (Preferred Reporting Items for Systematic Reviews and Meta-Analyses) method using 6 parameters (research approach followed, disease[s] diagnosed, dataset[s] used, algorithm[s] applied, performance achieved, and future direction[s]).

**Results:**

Initially, 437 articles were collected from 5 major groups of identified sources; 14 articles were selected for the final analysis. Results revealed that, compared with traditional methods, the MMDF methods improved model performances for the diagnoses of skin NTDs and non-NTD skin diseases. Algorithmically, convolutional neural network (CNN)–based models were the predominantly used DL architectures (9/14 studies, 64% ), providing feature extraction, feature fusion, and disease classification, which were also conducted with transformer-based methods (1/14, 7%). Furthermore, recurrent neural networks were used in combination with CNN-based feature extractors to fuse multimodal data (1/14, 7%) and with generative models (1/14, 7%). The remaining studies used study-specific algorithms using transformers (1/14, 7%) and generative models (1/14, 7%).

**Conclusions:**

Finally, this article suggests that further studies should be conducted about using DL-based MMDF methods for skin NTDs, considering model efficiency, data scarcity, algorithm selection and use, fusion strategies of multiple modalities, and the possible adoption of such tools for resource-constrained areas.

## Introduction

### Background

Neglected tropical diseases (NTDs) are the most prevalent diseases, affecting more than 1 billion people worldwide, as estimated by the World Health Organization (WHO), and particularly impact people living in the world’s tropical areas who are among the poorest, most vulnerable, and outcast groups [1]. They have a devastating impact on people’s physical, mental, and social well-being [1,2]. According to the WHO [2,3], NTDs represent 21 different diseases that include Buruli ulcer, Chagas, dengue and chikungunya, dracunculiasis, echinococcosis, foodborne trematodiases, human African trypanosomiasis, cutaneous leishmaniasis (CL), leprosy, lymphatic filariasis, mycetoma, chromoblastomycosis and other deep mycoses, noma, onchocerciasis, rabies, scabies and other ectoparasitoses, schistosomiasis, soil-transmitted helminthiases (STH), snakebite envenoming, taeniasis/cysticercosis, trachoma, and yaws.

Once a particular disease occurs, proper diagnostic measures should be in place. Although each disease has its own diagnostic procedures, most NTDs can be diagnosed using skin-related symptoms; 18 of the 21 NTDs recognized by the WHO have primary skin indicators or associated clinical features and are thus called skin NTDs [4,5]. The diagnosis of skin diseases relies on a visual examination of the skin of affected patients; therefore, the diagnosis of skin NTDs requires visual inspections of patients’ skin. This inspection can be enhanced by integrating technological interventions.

Current diagnostic research suggests that the diagnosis of skin NTDs can be enhanced using artificial intelligence (AI)–enabled diagnostic systems to overcome the challenges of these diseases [6]. On the other hand, the diagnosis of NTDs often involves a combination of different types of clinical data, possibly from diverse sources, such as patient medical records, visualization of patient skin, and laboratory tests. Hence, in light of current achievements, the use of AI-enabled diagnostic systems, particularly deep learning (DL)–based systems that incorporate multimodal data fusion (MMDF) techniques, has potential for the diagnoses and recognition of skin-related NTDs.

How are such systems developed to assist the diagnosis of skin NTDs? Answering this question seems quite difficult, as there are very few similar research-based systems that are publicly available to be able to analyze their approaches. Therefore, how can we approach this issue and analyze the methods, tools, and techniques used in previous studies to develop DL-based systems with MMDF for skin NTDs? To address this issue, we conducted a systematic review of previous studies applying MMDF techniques for skin NTDs and non-NTD skin diseases, since both groups of diseases can be diagnosed using common dermatological approaches to examining skin manifestations.

Although the specific diagnostic procedures to be used might be different, from the dermatological point of view, the general procedures for skin NTDs or non-NTD skin diseases remain the same: visual examination of the skin along with patient data processing [7]. Accordingly, in DL-based diagnostic research such as experimenting with MMDF for skin NTD diagnosis, particularly for leprosy [8], and using MMDF for multiple non-NTD skin diseases [9], the DL models learn to examine skins using clinical images (both cited studies used dermoscopic images) and integrate textual clinical data to predict the disease. This clearly shows that the MMDF methods used for non-NTD skin diseases can be adopted for skin NTDs because the 2 skin disease groups share the same general procedure.

Hence, this article presents a deeper analysis of DL-based MMDF techniques that are demonstrated in previous studies for the diagnosis of skin NTDs and non-NTD skin diseases using the PRISMA (Preferred Reporting Items for Systematic Reviews and Meta-Analyses) method. This approach was used to analyze the methods, data fusion techniques, algorithms, and related aspects used for specific diseases so as to demonstrate the potential of using those techniques for the diagnosis of skin NTDs. The studies analyzed in this review confirmed that the use of MMDF techniques outperformed traditional diagnostic models that implemented DL methods without MMDF [9-12]. In view of these facts, this study aimed to conduct a systematic literature review to provide a deeper analysis and appraisal of previous studies using the PRISMA method, guided by the following questions:

- What DL methods or approaches were used for the diagnosis of NTDs or non-NTD skin disease(s)?
- Which data fusion methods were used for skin disease diagnosis tasks?
- What types of medical data were integrated to demonstrate the MMDF method for the diagnosis of skin disease(s)?
- Which algorithms were used, and how did each algorithm perform in the proposed DL-based MMDF diagnostic model?

### DL and Diagnosis of NTDs

In real-world clinical settings, efficient disease diagnostic processes are basically carried out by using multiple types of medical data that are taken from different sources and different formats or modalities, including textual patient information and clinical images such as X-rays, dermoscopic images, or even patient skin images. The integrative use of diverse modalities of medical data can enhance the diagnostic processes, thereby enhancing the quality of health care services, by using machine learning (ML) algorithms, including DL methods. Technically, this process of integrating multiple modalities of data (possibly taken from different sources) is called MMDF [13,14].

MMDF techniques are playing vital roles in developing intelligent disease diagnostic systems for different diseases, such as in dermatology [15]. In this regard, MMDF techniques are advancing diagnostic accuracy, where these methods outperform baseline methods, as presented in [16]. Regarding the diagnosis of NTDs, including skin NTDs, there have been efforts toward the use of AI-based diagnostic tools built using ML approaches. In this regard, Ali et al [17] used ML methods for early prediction of schistosomiasis and concluded that the CatBoost model achieved the highest accuracy, at >80%. Furthermore, since skin NTDs are diagnosed using skin manifestations, deploying DL-based approaches for the diagnoses of these diseases could have the potential to support and enhance the diagnostic processes, as confirmed by recent studies (eg, [8,18,19]) that applied DL methods and algorithms using skin images for the diagnosis of skin NTDs. All the aforementioned studies used ML and DL methods to diagnose skin NTDs and achieved remarkable results in terms of diagnostic accuracy.

However, based on extensive exploration, we found that there were very few efforts toward the implementation of MMDF methods in AI-based diagnostic platforms for skin NTDs, except for 2 studies found very recently. To this end, Barbieri et al [8] conducted a study that demonstrated MMDF methods for the diagnosis of leprosy and claimed that they achieved higher accuracy in distinguishing leprosy (Hansen disease), which is one of the most widely spread NTD types globally, with 120,000 new cases being reported every year [20]. A recent study by Achary et al [21] implemented the MMDF method using a generative adversarial network (GAN)–enabled multimodal diagnostic framework using a recurrent neural network (RNN)–based architecture for merging multimodal data.

### Data Fusion Techniques and Approaches

#### Overview

Data or information fusion represents the use of data or information from different sources in different formats or modalities for interpretation in all tasks that require any type of parameter estimation or prediction using data or information [22]. Data fusion, including MMDF, is the process of combining different data streams that include different dimensionality, resolution, and types of data to generate information in a form that is more understandable by or usable to an end user or system [22,23]. Although the fusion techniques represent individual methods used to achieve the data fusion process, there are 3 main fusion techniques for integrating multimodal data: early fusion, joint fusion, and late fusion.

#### Feature-Level Fusion or Early Fusion

Feature-level fusion refers to the process of integrating multiple input modalities into a single feature vector before feeding into one single ML model for training, where the input modalities can be joined in many different ways, such as concatenation or pooling [24,25]. This fusion technique is suitable for combining similar feature sets at the early stage of model development if data of similar modalities are to be collected from different sources.

#### Intermediate Fusion or Joint Fusion

This type of fusion combines learned feature representations from intermediate layers of neural networks with features from other modalities as input to a ﬁnal model, and it is implemented with neural networks due to their ability to propagate loss from the prediction model to the feature extraction model(s) [26].

#### Model-Level Fusion, Decision-Level Fusion, or Late Fusion

Late fusion uses predictions from several models to make a ﬁnal decision, where different modalities are used to train separate models and the ﬁnal decision is made using an aggregation function to integrate the predictions of the different models. The aggregation functions might include averaging, majority voting, weighted voting, or a metaclassiﬁer based on the predictions from each model. The choice of aggregation function is usually empirical, and it varies depending on the application and input modalities [26,27]. This fusion technique is more like an ensemble learning method, where the abilities of different models are combined to make up for the biases and errors of a single model to achieve better performance [28].

#### Fusion Approach

Apart from the data fusion techniques used to actually merge different modalities of multimodal data, the fusion approach is a major aspect that defines the general perspective toward solving a given data fusion problem. The approaches can be described as presented in the following sections.

#### Feature Fusion

This is a data integration technique used to aggregate multiple feature sets extracted from multiple input data to generate a single feature set [24]. In image processing problems, it refers to the fusion of feature vectors of training images extracted from a shared weight network layer and feature vectors composed of other numerical data [25]. It helps to learn image features fully for the description of their rich internal information [29].

#### Model Fusion

Also known as late fusion, model fusion represents a fusion approach that combines different models. The study by AlDahoul et al [30] combined 2 deep neural networks (DNNs), including a binary normal/attack classifier and multi-attack classifier, to train a DNN for network anomaly detection. As mentioned by Shoumy et al [24], the model fusion technique uses the connection between experimental data under different modalities.

#### Image Fusion

Image fusion is a fusion approach that combines different images and generates informative images by integrating images obtained from different sources [31]. Wang et al [32] suggested that aggregating medical images helps to enhance diagnostic accuracy. This claim was demonstrated by fusing clinical images and dermoscopic images using deep convolutional neural network (DCNN) methods, which achieved an overall accuracy greater than 80%. Although clinical images are clinically captured photographs, dermoscopic images represent images taken by dermatologists using dermoscopy devices [33].

#### Multimodal Data Fusion

Multimodal data represent the different formats or modalities of data, such as text, image, video, and audio. An MMDF approach is used for combining particular modalities to derive multimodal representation [12,13,23,34]. This approach has multiple applications for health care systems, as it allows the combination of diverse modalities of data, such as the textual medical history and clinical images of patients (such as skin images from patients), to form a single multimodal dataset that can be used to train diagnostic models using ML and DL methods.

### Significance and Novelty of the Review

This study presents a systematic review regarding the use of DL-based MMDF techniques for the diagnosis of skin NTDs. However, the current literature reveals a lack of previous studies that have applied MMDF techniques for the diagnosis of skin NTDs, marking a critical gap. Hence, the study used an alternative approach by incorporating studies conducted on skin NTDs and non-NTD skin diseases, as both types of diseases are diagnosed using skin manifestations. Based on the gaps, this study analyzed studies that applied MMDF techniques, thereby presenting the first systematic review to our knowledge that analyzes methods using both skin NTDs and non-NTD skin diseases. Furthermore, the study identified a series of promising approaches to apply MMDF techniques for skin NTDs, all marking the novelty of this study. Overall, this review highlighted key current gaps and future research directions, which can motivate further research in the area.

## Methods

As the basis for conducting and reporting this systematic review, we followed the steps recommended by the PRISMA model [35,36], as described in the following sections.

### Search Strategy

To acquire all relevant articles regarding the application of DL-based MMDF methods for the diagnosis of skin NTDs, we conducted a comprehensive and systematic literature search by establishing a search strategy designed to maintain both depth and breadth. Accordingly, beginning with the identification and selection of relevant article sources, the search was performed across 5 different categories of sources, as described in the following sections.

#### Search Engine (Google Scholar)

Strategically, we started the search process using the free web-based search engine that has the broadest indexing coverage of scholarly literature, Google Scholar. We used this search engine for 2 major purposes: identifying a wide range of articles and supplementing major databases to be searched.

#### Bibliographic Databases

Primarily, we identified 2 major bibliographic databases based on 2 perspectives, the subject matter and problem domain of this study. First, as a medical-focused study, the PubMed database was selected and primarily used to find relevant articles that deal with the diagnosis of skin NTDs and non-NTD skin diseases using AI-based methods. Second, the web-based searchable bibliographic database, ScienceDirect (Elsevier), was included to extract more full-text articles. We selected these 2 databases because they have wide multidisciplinary coverage and robust indexing mechanisms.

#### Publishers

In addition to the databases, we conducted targeted searches of specific publishers’ platforms to make sure that there were no specialized articles remaining. Accordingly, we selected IEEE Xplore to specifically acquire articles in the area of technology, given that DL-based methods have wider applications for disease diagnosis, including skin NTDs and other skin diseases. MDPI (a publisher of open-access scientific journals and books) and Springer Nature were also selected.

#### Specialized Journals

We conducted targeted search operations of specific journals to ensure that we captured all the studies that focused on NTDs, and we included the following sources. African Journals Online (AJOL), a prominent source of literature on health and tropical medicine in Africa, was included to look for studies that are of specific relevance and research focus, as this study was based in Africa. Similarly, Tropical Medicine and Health and PLOS (as it specifically incorporates PLOS NTD) were included due to their direct relation to the topic of our study, and related studies were acquired from these journals.

#### Gray Literature Searching

This method allowed us to explore specific journals and nondatabase sources obtained from academic social networks using Mendeley (reference management software). Generally, all these sources were selected based on the broader indexing coverage of the search engine, the relevance of the databases to the research topic, the scope and reproducibility of this study, and the collaborative decision of the authors.

To complete the search process, search terms guided by the research keywords were formulated by choosing terms that are highly relevant to the core topics, contents of the articles, and objective of this study. Using the Boolean operators (“AND” and “OR”) to systematically combine the selected search terms, 6 different search terms were developed that include the following search terms: [(((((“Neglected Tropical Diseases”) OR (“NTDs”) OR (“skin related Neglected Tropical Diseases”) OR (“Skin-Related Neglected Tropical Diseases”) OR (“Skin NTDs”) OR (“tropical diseases”)) AND ((“Diagnosis”) OR (“diagnostic model”) OR (“classification model”))) AND ((“Deep Learning”) OR (“DL”) OR (“Convolutional Neural Network”) OR (“CNN”) OR (“Deep Neural Network”) OR (“DNN”) OR (“Recurrent Neural Network”) OR (“RNN”))) AND ((“Data Fusion”) OR (“Data Fusion Techniques”) OR (“Data Fusion methods”) OR (“Multimodal medical Data”) OR (“Multimodal Data Fusion”) OR (“Multimodal Data Fusion Techniques”)))]. The final set of search terms was used on each of the selected article sources to deeply search and filter relevant articles. The complete list of sources and the search terms prepared and used in each source are presented in Table S1 in Multimedia Appendix 1.

Finally, all the search results were exported to EndNote to create a library using titles of the articles to identify each article, which was used for relevant article selection performed in a 5-level screening process.

### Eligibility Criteria

Not all articles are critically relevant for the review concerning the integration of MMDF techniques based on DL methods for the diagnosis of skin-related NTDs. Hence, a set of inclusion criteria were applied. Articles were included that had an appropriate level of similarity and relationships in the topics and contents with the search keywords used to deeply search and filter the articles, demonstrated DL methods for the diagnosis of skin NTDs or non-NTD skin diseases with proper evaluation of the methods used, demonstrated proper use of MMDF techniques for the diagnosis of skin NTDs or non-NTD skin diseases, used privately collected and targeted datasets, and presented original research containing full-text content published in peer-reviewed journals or conference proceedings.

Similarly, a set of exclusion criteria were used. These criteria included articles that did not use DL-based MMDF methods, used DL-based MMDF methods for diseases other than skin diseases, were review articles and articles without full text, were published in languages other than English, and were published prior to the year 2014.

### Article Search

The 2 searching methods (“basic search” and “advanced search”) were applied to the selected article sources. First, the ordinary (basic search method) was used, in which general titles and the research keywords were entered in the regular search box of each of the data sources and searched. Second, the advanced search option was used with the proposed search terms, which allowed us to specify relevant options to include subject areas, related topics, and publication dates to obtain articles that were relevant to the topic by narrowing the search results. Using both of the search methods and search terms, the search was conducted in each of the selected article sources, search engine, bibliographic databases, publishers, and specific journals. By specifying article publication dates, articles published between the years 2014 and 2025 were collected and prepared for screening. Furthermore, literature sources outside the popular publishers and nondatabase sources, which included academic web portals, academic libraries, and research sites, were also consulted to add relevant content.

### Relevant Article Selection

The entire article selection process was conducted based on the PRISMA method, which is an evidence-based minimum set of items for reporting systematic reviews and meta-analyses [35,36]. The use of multiple sources, especially the search engine, created duplication of databases, journals, and article files. This required systematic screening procedures. We conducted the first-level screening based on external duplicate removal. External duplicates occur when the same article is acquired from different sources, especially when using search engines and different databases. Hence, for the first-level screening, the articles were screened, mainly in a manual approach using the titles. The next levels of screening were performed using software tools such as EndNote and Rayyan. As a reference management tool, we used EndNote to create a library of the collected articles, manipulate the articles, process the collected articles, and automatically deduplicate files in the library. Finally, for higher-level screening, Rayyan, the AI-based free online software tool [37], was used mainly to speed up the literature screening process based on title, author names, abstracts, study area, and full texts in the article library exported from EndNote.

### Ethical Considerations

As a review study, we followed strict ethical procedures throughout the entire process of this study, starting from problem formulation, article exploration, evaluation, and analysis, up to reporting. Furthermore, for the articles reviewed in this study, we analyzed the papers for potential ethical issues since all the articles reviewed used data collected from patients. This study did not involve any human or animal participants, clinical trials, or any data related to patients or clinical trials. Therefore, a specific ethical assessment was not sought for this study, as the study presented only the methodological analysis of previously published studies applying DL-based MMDF techniques for the diagnosis of skin NTDs and non-NTD skin diseases.

### Quality Assessments

For the quality assessment of the included studies, we used the QUADS-2 (quality appraisal for diverse studies) tool, which is recommended for use in systematic reviews to assess factors such as the risk of bias and applicability issues of diagnostic research studies. Assessments are carried out based on 4 domains, namely patient selection, index test, reference standard, and flow and time [38]. As a DL-related study involving medical diagnostics, using the QUADS-2 assessment tool, we evaluated the selected studies for potential biases that might arise from the methods related to patient selection and algorithmic biases.

## Results

### Collected Articles

**Figure 1:**
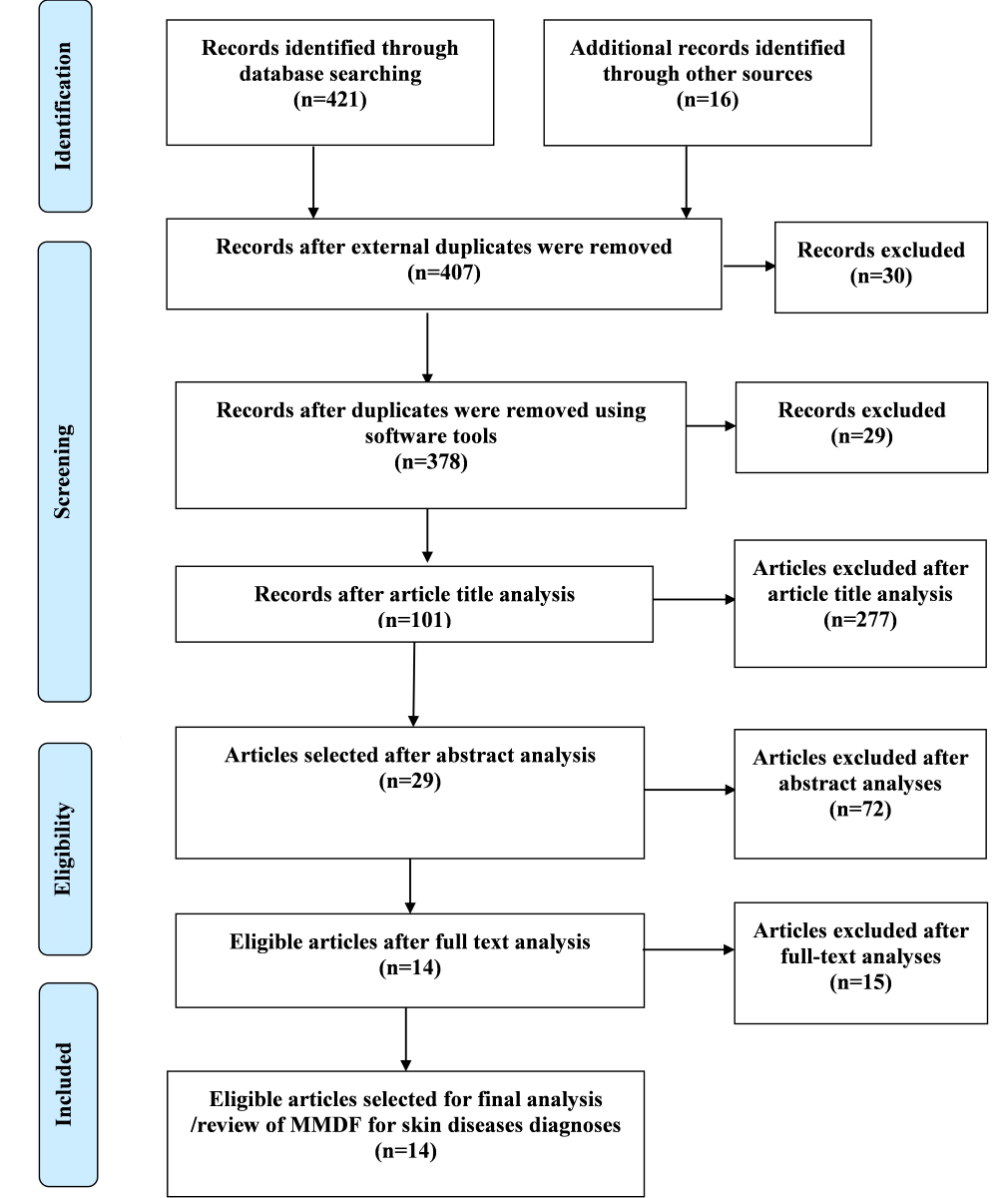
Article selection for systematic literature review following the PRISMA (Preferred Reporting Items for Systematic Reviews and Meta-Analyses) flowchart. MMDF: multimodal data fusion.

Overall, 421 articles were acquired from the 7 major sources identified (databases, publishers, and specific journals) and the search engine, while 16 articles were collected from the gray literature search. We then implemented a series of screening operations on the collected articles in order to identify the most relevant set of articles. The first-level screening resulted in 30 articles being screened out as a result of external duplicates, which allowed us to select 407 articles. Second, the use of EndNote software resulted in the removal of 27 duplicate articles, as the articles were acquired from 10 different sources (5 categories, including the search engine) organized in different folders. The automatic deduplication process continued in Rayyan, which identified 2 pairs of duplicated articles and removed 2 of them, resulting in 378 unique articles. The overall article selection process is outlined using the PRISMA flowchart in Figure 1.

For the third-level screening, the online software tool Rayyan was further used to screen based on article title, and in the process, 101 articles that had a direct relationship with the current topic of the study were selected. The fourth-level screening was conducted using abstract analysis, and 29 articles were identified. Finally, only 14 articles were found to be eligible for the final analysis after full-text analysis to check eligibility of the 29 articles identified during the fourth round of screening.

### Distribution of Articles

The application of the specified searching methods in the selected sources resulted in 437 articles published between the years 2014 and 2025, as shown in Table 1, depicting the distribution of the collected articles based on their sources. As shown, Google Scholar allowed us to collect 188 articles (188/437, 41.2%) from 9 different sources. These included PubMed, ScienceDirect, IEEE Explore, MDPI, Springer Nature, AJOL, NCBI, PLOS, and Tropical Medicine and Health, creating duplication of sources (databases, publishers, and journals).

Further analysis was performed regarding the sources of the articles with respect to the first 2 initial levels of screening, as shown in Table 1. From the 437 articles initially collected, the first-level screening allowed us to select 407 articles by excluding 30 articles. We further excluded 29 duplicate articles during the second level of screening by merging the sources into one big library in EndNote, resulting in 378 articles to be further screened.

**Table 1:** All collected articles and their distribution by article databases or search engine (N=437).

Source	Articles initially collected, n (%)	Articles after first-level screening (n=407), n (%)	Articles after second-level screening (n=378), n (%)
Google Scholar	188 (43)	173 (42.5)	166 (4.4)
IEEE Explore	68 (15.6)	61 (15)	55 (14.6)
ScienceDirect (Elsevier)	49 (11.2)	49 (12)	42 (11.1)
Springer Nature	45 (10.3)	43 (10.6)	38 (10.1)
MDPI	37 (8.5)	33 (8.1)	33 (8.7)
Mendeley	21 (4.8)	20 (4.9)	17 (4.5)
PubMed	13 (3)	13 (3.2)	13 (3.4)
Other	16 (3.7)	15 (3.7)	14 (3.7)

### Distribution of Articles by Publication Year

During the third level of screening, 101 articles relevant to the topic and published between the years 2014 and 2025 were selected using title analysis, as shown in Table 2.

As shown, the selected articles included recent studies, with 3% (3/101) published in 2025 and 2% (2/101) published in 2024. The majority of the studies were published in 2023 (30/101, 29.7%).

**Table 2:** Distribution of articles after the third-level screening by publication year (n=101).

Year	Results, n (%)
2014	1 (1)
2015	1 (1)
2016	1 (1)
2017	1 (1)
2018	4 (4)
2019	3 (3)
2020	15 (14.9)
2021	14 (13.9)
2022	26 (25.7)
2023	30 (29.7)
2024	2 (2)
2025	3 (3)

### Distribution of Articles by Methods Used

Finally, the 101 articles were further analyzed based on their research approach, which represents the main analysis parameter of this study. This analysis categorized the studies into 6 groups according to the specific methods, tools, and techniques used. The first group included articles using DL-based MMDF methods for the diagnosis of skin NTDs, representing 2% (2/101) of the selected articles. The second group included articles that implemented only DL-based methods for the diagnosis of skin NTDs, representing 6.9% (7/101) of the selected articles. The third group included articles that implemented DL-based methods for the diagnosis of non-skin NTDs, representing 4% (4/101) of the articles. The fourth group comprised articles that used DL-based MMDF methods for the diagnosis of non-NTD skin diseases, representing 20.8% (21/101) of the selected articles. The fifth group included articles that used only DL-based methods for non-NTD skin diseases, representing 48.5% (49/101) of the selected articles. Finally, the last group included articles that demonstrated other forms of data fusion methods, representing 17.8% (18/101) of the articles.

Accordingly, 87.1% (88/101) of the selected articles used DL-based methods for non-NTD skin diseases, and 48.5% (49/101) used DL methods for the same non-NTD skin diseases. The remaining 20.8% (21/101) of the articles used MMDF for non-NTD skin disease diagnoses, and 17.8% (18/101) of the articles used other forms of fusion, such as feature fusion, model fusion, and image fusion. Of the selected articles, 12.9% (13/101) applied DL-based methods for the diagnosis of NTDs in general, of which 4% (4/101) implemented DL-based methods for the diagnosis of non-skin NTDs (NTDs that have no skin manifestations such as STH [39]). However, none of the articles implemented MMDF techniques, apart from recommending MMDF for future work, like in [19]. Only 2% (2/101) of the articles applied DL-based MMDF methods for the diagnosis of 2 different skin NTDs, leprosy [8] and malaria and dengue [21]; only dengue is a skin NTD. The remaining articles presented reviews and mixed approaches of DL-based methods for NTD diagnosis and were hence excluded from the final analysis. These results showed that the MMDF method has not been used to its potential for AI-based diagnosis of skin NTDs. Table 3 presents the articles demonstrating DL-based methods for skin-related and non-skin NTDs.

**Table 3:** Review of the deep learning (DL)–based methods for skin neglected tropical diseases (skin NTDs) and non-skin NTDs.

Citation		Publication year	Study approach	Disease(s) diagnosed	Dataset(s) used	Algorithm(s) used	Performance achieved
**Skin NTDs**							
	[6]	2023	Clinical photo, transfer learning	5 skin NTDs	1709 images from 506 patients	CNN^a^ (VGG16, ResNet50)	ResNet50 accuracy (Tasks 1 and 2: 84.63% and 84.17%, respectively)
	[19]	2022	Data collection, preprocessing, SVM^b^ optimized by BHO^c^	Buruli ulcer, CL^d^, and leprosy	1054 images (420 Buruli ulcer, 262 CL, 372 leprosy)	SVM, BHO	Accuracy, *F*_1_-score, recall, and sensitivity: 96%, 89%, 90%, and 92%, respectively
	[18]	2023	Siamese-based FSL^e^, metalearning on small dataset	Leprosy	368 leprosy, 28 nonleprosy, 151 patients	Siamese network, HAC^f^	FSL accuracy (training: 91.25%; testing: 73.12%)
	[40]	2024	DL method using clinical photos	5 skin NTDs	1709 images of 506 patients (data store)	CNN (VGG16, ResNet50)	Accuracy: 70%; ResNet50 higher than VGG16
**Non-skin NTD**							
	[39]	2022	WSI^g^, KK^h^ stool thick smears	STH^i^	16,990 annotated helminth eggs	CNN (ResNet101 COCO)	Precision: 94.9%; recall: 96.1%
	[41]	2021	Crowd-sourced annotated images	STH	41 digitized stool samples (10,319 cropped images)	CNN (MobileNet-V2)	AUC^j^ (crowd: 0.728; expert-annotated: 0.932)
	[42]	2024	Digitization of slides, visual analysis, KK method	STH	Stool samples (n=1335) collected from school children	CNN (ResNet50), YOLOv2	High sensitivity (80%) and specificity (98%) for *Ascaris lumbricoides*

^a^CNN: convolutional neural network.

^b^SVM: support vector machine.

^c^BHO: black hole algorithm.

^d^CL: cutaneous leishmaniasis.

^e^FSL: few-shot learning.

^f^HSL: hierarchical agglomerative clustering.

^g^WSI: whole slide imaging.

^h^KK: Kato-Katz.

^i^STH: soil-transmitted helminthiases.

^j^AUC: area under the curve.

As presented, the study by Yotsu et al [6] demonstrated the use of a DL-based diagnostic model for NTDs using skin images only and achieved 70% accuracy. Beesetty et al [18] conducted a study for leprosy skin lesion detection using a Siamese neural network–based few-shot learning model and applying a hierarchical agglomerative clustering algorithm for a small clinical dataset and reported an accuracy of 73.12%. An optimized diagnostic approach was also proposed for NTDs by selecting 3 diseases and developing a model using support vector machine (SVM) and the black hole algorithm, achieving more than 90% accuracy [19]. A recent study [40] reported the creation of a DL model based on CNN architectures (VGG16, ResNet50) using clinical photos obtained from a previous data source to train the model. The studies of non-skin NTDs proposed DL models for the diagnosis of STH using images collected through different methods that included Kato-Katz, stool thick smears, and whole slide imaging [39,41,42].

### Analysis of Fusion Techniques Used

#### Overview

Table 4 presents the review of the fusion methods other than MMDF techniques used by studies.

As shown, 5% (5/101) of the articles used feature fusion techniques, while 1% (1/101) of the articles implemented the image fusion technique for the diagnosis of non-NTD skin diseases. Since the fusion methods used and diseases diagnosed did not directly fit into the objective of this study, these 5.9% (6/101) of studies were excluded from the final analysis. Finally, 13.9% (14/101) of the eligible articles were selected for the final analysis.

The 14 eligible articles used DL-based MMDF techniques for the diagnoses of skin NTDs and non-NTD skin diseases. Accordingly, 14% (2/14) of the articles implemented DL-based MMDF techniques for the diagnosis of 2 skin NTDs (leprosy and dengue), while 86% (12/14) of the articles described MMDF techniques for non-NTD skin disease diagnoses such as skin cancer, skin lesions, and other types of skin conditions. Table 5 presents a summary using 3 major parameters (method[s] used, algorithm[s] used, and corresponding performance achievements). The complete analysis is provided in Table S2 in Multimedia Appendix 1.

**Table 4:** Review of fusion methods other than multimodal data fusion (MMDF) for skin disease diagnoses.

Citation	Publication year	Study method or approach used	Disease(s) selected	Dataset(s) used	Algorithm(s) used	Performance achieved
[43]	2022	Multiclass skin lesion classification using feature fusion and ELM^a^	Skin disease (skin lesion)	HAM10000^b^ and ISIC^c^ 2018	SVM^d^, KNN^e^, DT^f^, NB^g^, ensemble tree, single hidden layer	Registered best accuracy of 94.36%
[32]	2021	Image fusion (clinical and dermoscopic): multilabeled deep feature	Skin cancer (melanoma)	Publicly available dataset	DCNN^h^, CC^i^, PCA^j^	Reported 81.3% accuracy
[44]	2019	Transfer learning and multilayer feature fusion network	Skin lesion	HAM10000 dataset	CNN^k^	High recognition (ROC^l^-AUC^m^ 96.51)
[45]	2022	Applied feature fusion for manual and automatic feature extraction	Skin cancer	DermIS dataset	CNN, LSTM^n^, LBP^o^, Inception V3	Achieved maximum accuracy of 99.4%
[46]	2023	Dual-branch (feature) fusion network using DCNN, transformer branches	Skin disease (skin lesion)	Private dataset XJUSL	DCNN	Reducing parameters (11.17 million) improved accuracy by 1.08%
[47]	2023	Feature fusion: FBB^p^, HFE^q^, and VGG19-based CNN	Skin cancer (melanoma)	ISIC 2017, academic torrents	CNN	Registered 99.85% accuracy

^a^ELM: extreme learning machine.

^b^HAM: human against machine.

^c^ISIC: International Skin Imaging Collaboration.

^d^SVM: support vector machine.

^e^KNN: k-nearest neighbors.

^f^DT: decision tree.

^g^NB: naive Bayes.

^h^DCNN: deep convolutional neural network.

^i^CC: classifier chain.

^j^PCA: principal component analysis.

^k^CNN: convolutional neural network.

^l^ROC: receiver operating characteristic.

^m^AUC: area under the curve.

^n^LSTM: long short-term memory.

^o^LBP: local binary pattern.

^p^FBB: fast-bounding box.

^q^HFE: hybrid feature extractor.

**Table 5:** Summary of the review of the deep learning (DL)–based multimodal data fusion techniques for the diagnosis of skin disease.

Citation	Study method or approach used	Algorithm(s) used	Performance achieved
[8]	New dataset (skin lesion images, clinical data, demographic data), model training, and algorithms (elastic-net LR^a^, XGB^b^, and RF^c^) to combine image analysis with metadata	XGB, RF, LR, and 3 different models used (Model 1 using Inception-v4, ResNet-50), elastic-net LR model integration	Model 1: moderate (best accuracy of 66.6% and AUC^d^ of 74.56%, elastic-net LR on metadata); Model 2: higher accuracy of 90%, AUC of 96.46%; RF on patient information achieved highest AUC of 98.74% on the testing patients
[9]	Multiplication-based data fusion using the metadata	CNN^e^, the color constancy algorithm	Outperformed traditional baseline methods (*P* values<.5)
[12]	DNN^f^-based multimodal classifier (wound images and their locations): body map development, multimodal network	AlexNet + MLP^g^, AlexNet + LSTM^h^, ResNet50 + MLP, VGG16 + LSTM	Maximum accuracy on mixed class varied from 82.48% to 100%; maximum accuracy on wound class varied from 72.95% to 97.12% in various experiments.
[21]	GANs^i^ and ensemble of BiLSTM^j^, BiGRU^k^, and RNN^l^ models for classification and including an experimental evaluation	GANs, ensemble of BiLSTM, BiGRU, RNN, and CRNN^m^	Improved performance (precision by 4.9%, accuracy by 3.5%, recall by 3.5%, and AUC by 4.5%)
[48]	Combining multiple imaging modalities (dermatoscopic and macroscopic) with patient metadata	CNN: 2 ResNet-50 architectures, RF classifier	Binary (AUC 0.866 vs 0.784) and multiclass classification (mAP^n^ 0.729 vs 0.598)
[10]	Combining images and metadata features: the (MetaBlock)	CNN using 5 pretrained models	Performed better than the other combination methods in 6 of 10 scenarios.
[11]	Performance analysis of classifiers and a naive combination of patient data and an image classifier	CNN	CNN: AUROC^o^ of 92.30% (SD 0.23%) and balanced accuracy of 83.17% (SD 0.38%); naive strategy: accuracy of 86.72% (SD 0.36%)
[49]	DNN with 2 encoders and application of a multimodal fusion module with intramodality self-attention and intermodality cross-attention	CNN: CNN models (ResNet-50)	Accuracy: 0.768 (SD 0.022); balanced accuracy: 0.775 (SD 0.022); outperformed other metadata fusion methods (MetaNet, *P*=.035; MetaBlock, *P*=.028)
[50]	ViT^p^ model, SLE^q^, and MA^r^ block	CNN: ResNet101, Densenet121, and ViT	Private dataset (accuracy: 0.816) and ISIC^s^ 2018 (accuracy: 0.9381; AUC: 0.99)
[51]	Medical image analysis: preprocessing, feature extraction, classification or diagnosis, and the hold-out technique to split the dataset	CNN: 6 pretrained CNN models, HPO^t^ algorithms	Average accuracy, sensitivity, specificity, precision, and DSC^u^: 9.94%, 91.48%, 98.82%, 97.01%, and 94.00%, respectively
[52]	Fusion of clinical skin image and patient clinical data, feature extraction, and attention mechanisms	CNN: VGGNet19, ResNet50, DenseNet121, and Inception-V3	Accuracy: 80.42% (improvement of about 9% compared with the model accuracy using only medical images)
[53]	TFormer^v^: transformer feature extraction backbone, MTP^w^ block	Swin transformer, MLP, MTP block	Average accuracy of 77.99% and diagnostic accuracy of 80.03% on the Derm7pt dataset
[54]	Skin cancer prediction (clinical metadata and dermoscopic images), transfer learning (EfficientNetB3), and TabNet	EfficientNetB3, TabNet, and attention-based fusion	Consistently higher accuracy (98.69%) for classifying skin cancer on multiple datasets
[55]	2 distinct LLMs^x^ (GPT^y^-4-turbo and Gemini-Pro-1.5) for generating clinical notes using cross-evaluation and consensus scoring methods (metrics of BLEU^z^ score, ROUGE^aa^ score, overlap coefficient, and Jaccard index)	ResNet-50, multimodal models (BERT^ab^-ResNet and ALBEF^ac^), and LLMs (for synthetic clinical note generation)	GPT-4-turbo outperformed Google Gemini Pro 1.5 at generating detailed and contextually rich clinical notes, with higher BLEU and ROUGE scores (0.87 and 0.84, respectively, compared with 0.81 and 0.78, respectively, for Gemini Pro 1.5)

^a^LR: logistic regression.

^b^XGB: eXtreme gradient boost.

^c^RF: random forest.

^d^AUC: area under the curve.

^e^CNN: convolutional neural network.

^f^DNN: deep neural network.

^g^MLP: multilayer perceptron.

^h^LSTM: long short-term memory.

^i^GANs: generative adversarial networks.

^j^BiLSTM: bidirectional long short-term memory.

^k^BiGRU: bidirectional gated recurrent unit.

^l^RNN: recurrent neural network.

^m^CRNN: convolutional RNN.

^n^mAP: mean average precision.

^o^AUROC: area under the receiver operating characteristic.

^p^ViT: vision transformer.

^q^SLE: soft label encoder.

^r^MA: mutual attention.

^s^ISIC: International Skin Imaging Collaboration.

^t^HPO: hyperparameter optimization.

^u^DSC: disc similarity coefficient.

^v^TFormer: throughout fusion transformer.

^w^MTP: multimodal transformer postfusion.

^x^LLMs: large language models.

^y^GPT: generative pretrained transformer.

^z^BLEU: Bilingual Evaluation Understudy.

^aa^ROUGE: Recall-Oriented Understudy for Gisting Evaluation.

^ab^BERT: bidirectional encoder representations from transformers.

^ac^ALBEF: align before fuse.

#### Methods Used for Building Diagnostic Models

According to the results, 79% (11/14) of the articles primarily used CNN-based DL architectures, 14% (2/14) of the articles implemented transformer-based networks, and the remaining 7% (1/14) of the articles implemented an RNN-based architecture. The CNN-based studies used DCNN architectures such as VGG16, VGGNet19, ResNet50, ResNet101, DenseNet121, Inception-V3, AlexNet, and EfficientNetB3. Accordingly, for an accurate detection of leprosy [8], Inception-V4 and ResNet50 models were used for image analysis along with random forest (RF), eXtreme gradient boost (XGB), and Elastic-Net logistic regression (LR) for text-based tabular data analysis, with Elastic-Net LR being a regularization algorithm. For more than 2 modalities of data, such as 2 different image types and textual data, the use of separate CNN architectures such as ResNet-50 with a classifier model such as RF was used to build fusion models [48]. For images in a dataset having different sizes, a group of CNN architectures of AlexNet, VGG, ResNet, DenseNet, and SENet (Squeeze-and-Excitation Network) were used to extract visual features from 300 × 300-pixel images while using progressive neural architecture search network (PNAS) architecture for 224 × 224 sized images [9]. The diverse CNN architectures were also used with other algorithms such as VGG16 and multilayer perceptron (MLP), VGG19 and MLP, VGG16 and long short-term memory (LSTM), VGG19 and LSTM [12], or other combinations of the CNN architectures paired with other algorithms (like SVM, RF, and XGB) to extract textual and visual features in parallel. Transformer-based architectures, such as using the ResNet101 and DenseNet121 models with vision transformers (ViT) models and multimodal transformers with MLP, were also used to build MMDF models [50]. Furthermore, a hierarchical ViT architecture (for feature extraction), MLP, and a fusion architecture with a hierarchical multimodal transformer were used as one potential approach [53]. The other DL method used was the RNN-based architecture implementing an ensemble of RNN models using bidirectional LSTM (BiLSTM) for textual feature extraction, bidirectional gated recurrent unit (BiGRU) for image analysis, and a standard RNN layer for combining the extracted features from BiLSTM and BiGRU models [21] for skin NTD diagnosis.

#### Fusion Strategies Suggested for Skin Disease Diagnosis

The studies reviewed demonstrated 4 different fusion approaches that included feature fusion, model fusion, image fusion, and MMDF techniques, where 93% (13/14) of the studies we appraised applied MMDF approaches for integrating mainly clinical images and textual medical data. One study (1/14, 7%) used the MMDF approach to combine 3 different modalities of data: 2 imaging modalities (dermatoscopic and macroscopic images) with patient metadata [48].

In the study of leprosy diagnosis [8], the late fusion (decision-level fusion) method was applied using 2-step patient-level models using, first, a CNN architecture to predict the probability of leprosy based on skin lesion images (Model 1); second, ML algorithms (elastic-net LR, XGB, RF) to predict the probability of leprosy based on metadata (Model 2); and third, a combination of the outputs from both models given to a third model (Model 3) to train and predict the final output. The other study applied feature-level fusion (early fusion) by outlining a structure that used BiLSTM and BiGRU models for extracting multimodal features (image and text), followed by an RNN architecture to merge the feature representations from BiLSTM and BiGRU models and predict the final output (predicting the probability of malaria or dengue) [21]. An attention-based metadata processing block (MetaBlock), which uses metadata for enhancing features extracted from the images throughout the classification, can be another multimodal fusion strategy [10]. The naïve approach [11], combining patient data and the image classifier by replacing the image classifier with the patient data classifier on slides with low output scores for a whole slide image classifier module, is another fusion strategy described. Similarly, the feature concatenation method was used to develop a wound classifier multimodal network by concatenating the image classifier and location-based classifier outputs [12]. A late fusion approach can also be adopted to integrate multiple modalities of data (2 image modalities) with textual data [48], where image feature vectors created from each feature extraction network were merged with the feature vector of the textual data. The other MMDF approach, inspired by the SENet network operation, was the use of a multiplication-based approach where feature importance was controlled by the metadata [9].

The other MMDF strategy is the transformer-based approach. Accordingly, a neural network with a multimodal transformer consisting of 2 encoders for feature extraction (image and metadata features), 1 decoder (to extract image features and fuse the multimodal information using the ViT model), a soft label encoder for the metadata, and a mutual attention block to fuse the different features [50], was used and showed promising results.

Similarly, a DNN with 2 encoders for extracting image and textual features, an MMDF module with intramodality self-attention and intermodality cross-attention capabilities, was used in [49], and the model outperformed other fusion models. In another study, a fusion system was developed using 4 procedures consisting of preprocessing the image and metadata, feature extraction using 6 pretrained models, feature concatenation (using CNN through convolutional, pooling, and auxiliary layers), and classification of skin disease [51]. In another study [52], a skin cancer diagnostic model was developed following 3 procedures: extracting features (skin images and patient clinical data using CNN architectures), using the attention mechanism (for handling the multimodal features), and developing a feature fusion model. The “divide and conquer” approach using transformers, as in [53], was the other MMDF strategy where a hierarchical multimodal transformer block was used to fuse multimodal image modalities (dermoscopic and clinical images), followed by a multimodal transformer postfusion block that used a cross-attention mechanism to combine the fused image features with textual patient data. The use of an attention-based fusion mechanism can be used to merge image features extracted by CNN architecture and clinical data processed by TabNet [54]. Another advanced fusion strategy can be the use of a dual-encoder architecture using separate models for extracting multimodal features (ResNet-50 for image analysis and bidirectional encoder representations from transformers [BERT] for text processing), followed by a joint-encoder architecture containing a mode that processes text and image data in parallel but, importantly, aligns these modalities before fusion [55].

#### Achievements of the MMDF Techniques for Diagnosing Skin Diseases

As the results are confirming, MMDF techniques outperform traditional baseline diagnostic approaches [9,10]; the majority of the studies reported that their proposed disease classification models achieved more than 80% accuracy, as reported in [8,10,11,48,50]. The use of the GAN-based framework for data augmentation to add additional training data improved the model accuracy by 3.5% compared with the traditional models experimentally trained by the same researchers [21]. The medical image analysis study based on feature extraction, feature concatenation, and classification methods [51] reported 99.94% accuracy in the classification of 7 selected skin diseases. Finally, the study that used 2 DNN-based encoders with multimodal fusion modules (intramodality self-attention and intermodality cross-attention) [49] reported a 76.8% accuracy.

### Article Quality Assessments

After evaluating the quality of the articles, potential sources of bias were identified based on the 3 major categories of risk factors, namely data collection, dataset used, and development methodology used by the studies. Accordingly, 6 studies [8,11,12,21,48,50] used datasets collected from a particular population, which shows the potential risk of selection bias. In addition, the dataset sizes used in these studies were relatively small, leading to a potential risk of bias regarding model generalizability. The other 6 studies [9,10,49,51-53] used imbalanced datasets with an expected risk of overfitting, which leads to algorithmic bias. Finally, the use of synthetic data, as in [8] and [55], introduce a risk of bias due to previous latent biases inherent to the generative models used.

## Discussion

### Goal of the Study

The primary goal of this study was to present a systematic review of previous studies demonstrating DL-based MMDF techniques for the diagnosis of skin NTDs. However, led by the scarcity of articles, similarity of the diagnostic procedures used for both skin NTDs and non-NTD skin diseases, similarity of methods used, and results reported by the studies, this review focused on studies that used MMDF for diagnosing both skin NTDs and non-NTD skin diseases. In doing so, articles were collected and analyzed, and results obtained from the analysis were interpreted, as presented in the following sections.

### Important Findings

#### Article Selection and Distribution

It was challenging to find publicly accessible articles on DL-based MMDF approaches for the diagnosis of skin NTDs, except for 2 recently published articles. The pathological similarity of the disease categories (both are dermatological cases) forced this review to focus on studies that used MMDF for the diagnoses of both skin NTDs and non-NTD skin diseases.

#### DL-Based Methods and the Preference for CNN

The use of CNN architectures by the majority of the studies (11/14, 79%), primarily for feature extraction and classification tasks, proved the effectiveness of CNNs at processing and analyzing multimodal data for skin-related disease diagnoses. The transformer-based architectures, through the use of ViT, attention-based mechanisms, and GAN-enabled augmentation methods, showed model accuracy improvements when used in MMDF systems for skin disease diagnosis.

Regarding the availability of diverse fusion techniques, the studies demonstrated different fusion techniques that included feature fusion, model fusion, image fusion, and MMDF.

Regarding the superior diagnostic accuracy of MMDF techniques, the MMDF techniques consistently outperformed the traditional, mostly single-modality diagnostic methods, as confirmed by the analyzed studies that reported superior classification accuracies.

### Interpretations of Results

This systematic review of articles presented important dimensions in the area of DL-based MMDF methods, and the use of the PRISMA model made the article selection process more systematic and transparent.

Hereunder, we present methodological implications of applying MMDF for skin disease detection.

### DL Methods and Architectures

Generally, the entire MMDF process can be achieved using 4 different DNN modules that perform data preprocessing, feature extraction, feature concatenation or combination, and disease classification, where feature extraction is the common task for all MMDF tasks. Hence, proper feature extraction models and tools should be used for the feature extraction tasks. In DL, CNN-based pretrained models such as ResNet50/101, VGG16/19, DenseNet, Inception, and similar DCNN architectures are the tools to use for image feature extraction. Similarly, the RNN architectures (such as LSTM, GRU, and standard RNN, including the bi-directional architectures [BiLSTM, BiGRU, RNN]) can also be the right tools to extract features, especially for sequential data like text, audio, and video. Furthermore, for larger textual data, large language models (LLMs) such as BERT and GPT can be used to contextualize the text data and extract features from the text data.

We found that CNN models are the predominant DL architectures, mainly due to their convenience and widespread use of CNNs, which resulted from the ability of CNNs to provide end-to-end learning and their ability to work on raw data without having prior knowledge [56]. However, although CNNs are effective for analyzing image-based data and RNNs are suitable for sequential and time series data, the choice of a particular DL architecture should be based on the type of input data from which the features are extracted. Thus, for feature extraction of input data representing multiple modalities (like image, text, audio, and video), using the combination of CNN and RNN architectures represents the proposed approach. The use of this approach for MMDF problems allows the MMDF models to use properly extracted spatial representations from CNNs as well as properly analyzed sequential or temporal representations from RNNs, leading to higher model performance. In addition, the use of other ML algorithms (RF, LR, MLP, XGB, and others like TabNet) with the DNN architectures will also contribute to performance achievements of MMDF models, as each algorithm has operational sensitivity for a particular data type other than image data. For instance, the RF algorithm performs well on textual data, XGB performs well on structured tabular data, and the LR algorithm can perform well on continuous numerical data.

### Fusion Techniques and Approaches

One important observation regarding MMDF is the level at which the MMDF process takes place. The application of late or model fusion techniques for the diagnosis of skin-related diseases, such as skin NTDs, can be considered the potential fusion approach, though it requires further investigation using sufficient quality data with sufficient computing resources to demonstrate ensemble learning techniques. The intermediate level is a more appropriate level for fusing multimodal data, where a separate DNN network is used for individual feature extraction from each data modality. A separate layer concatenates the outputs of each network by combining the feature vectors of each modality from each network, followed by final processing layers such as pooling, normalization, and output layers to produce efficient predictions. This approach ensures effective data preprocessing by using a dedicated CNN architecture for modality-specific feature extraction targeting skin images and textual patient records. It also helps with creating bigger feature maps containing important features extracted from individual data modalities, which can improve the prediction accuracy of the diagnostic models. However, this technique is tedious, as it requires the development of an individual CNN model for each data modality, followed by another model for concatenation and prediction. Technically, most DL-based feature fusion tasks are performed at the intermediate level of data fusion. As a result, a potential problem might arise due to the addition of more features or dimensions that increase the complexity of the data, the curse of dimensionality. It is a problem resulting from high-dimensional data where the data have a large number of features, variables, or dimensions, often represented by the columns in a dataset [57]. To overcome this challenge, it is imperative to consider proper dimensionality reduction methods such as principal component analysis, which simplifies the existing variables to reduce the number of variables without losing the information contained in the initial data [58].

Regarding the fusion approaches, experiments are showing that prospective ML approaches, such as attention-based mechanisms and multimodal transformers, are demonstrating promising results for integrating multimodal data with the enhanced accuracy of DL-based diagnostic systems with multimodal data. In this regard, the attention mechanisms, such as the self-attention and cross-attention mechanisms, are showing promising results, as these techniques enable the capture of pertinent information from feature maps. This mechanism allows assigning different weights to different features, where features with larger weights are considered to be more important in the diagnostic process and vice versa, ensuring up to 2% performance superiority over other MMDF methods [49]. On the other hand, the use of multimodal transformers using the ViT model is another possibility that needs further investigation. Although these models outperform previous models in skin disease diagnosis by about 1%, unlike CNNs, ViTs are mostly data-intensive and require training models on public datasets with millions of labeled data [50]. In a DL-based MMDF application scenario for skin-related disease diagnosis, the integration of LLMs with image analysis models is one potential application. This method allows the integration of image data with nontabular textual patient data, such as disease symptoms expressed in longer phrases or sentences and clinical notes. LLMs, such as BERT and GPT, can be used to extract features from textual data to be concatenated with other modalities of data such as images. Finally, as a potential future trend, a hybrid fusion approach using a combination of more than 2 data modalities and different fusion approaches can be an alternative approach, although deeper investigation with multiple datasets is required to confirm this claim.

### Dataset Quality and Quantity

As a DL problem, the performances of the models can be impacted by data scarcity and the use of low-quality datasets, such as image resolution limitations, problems with annotation accuracy, and possible data imbalance among the skin diseases. Among the reviewed articles, 64% (9/14) of the studies used publicly available datasets; hence, issues of dataset sizes and data imbalances were not faced. Data scarcity was the major issue faced by the remaining 36% (5/14) of the studies that used private datasets specifically collected for the proposed diagnostic models. The problem of data scarcity can be addressed using an ML method called data augmentation, which can be used to artificially create data samples to increase the size and diversity of the dataset. The data augmentation process can be achieved in different ways, one of which is to implement data augmentation methods using built-in augmentation libraries during model training. The other method can use GAN-based generative models to increase dataset sizes through the creation of artificially synthesized data samples of a given data modality. The GAN-based method can be used as a data augmentation method applied both to the images and the clinical data [21]. The generative models also provide ways to generate artificially synthesized content such as text files, as in [55], for creating artificial clinical notes that were not part of the initial dataset, allowing them to address the problem of multimodal alignment. Multimodal alignment is a data modality imbalance problem that occurs when some data modalities (image, text, or video) appear to be rare, scarce, or unavailable, possibly due to difficulty acquiring them [59]. Hence, the GAN and LLM-based generative methods are potential solutions for maintaining dataset quality, quantity, and multimodal alignment. However, the use of these models, though critically useful in data-scarce scenarios, should be conducted with great care, giving attention to possible model performance issues when faced with real-world data.

### Study Limitations

The major constraints of this study are attributed to the methodology and resource limitation problems. First, the lack of previous studies on the application of MMDF techniques for skin NTDs broadened the scope of this review to include studies that focused on non-NTD skin diseases. Although this approach introduced a minor methodological misalignment, it allowed the inclusion of 12 selected articles to extend the analysis on a broader range of MMDF techniques. Second, due to resource limitations, the study was designed to explore and analyze open-access articles only. This has limited the number of relevant articles being collected and analyzed, which might have an impact on the overall analysis of results. Third, the vastness of the problem has also had negative impacts on the full contribution of this review study due to time constraints. Therefore, to confirm these results, future work should expand the scope of the analysis by conducting large-scale, targeted investigations using a broader range of analytical parameters.

### Article Quality Assessments

As a model development study, each study specifies the processes involved in the development and evaluation of the corresponding proposed diagnostic model for the identified disease. Accordingly, the potential issues observed in relation to the risk of bias mostly arise from the data collection, dataset creation, and model development processes leading to the issues of patient selection, dataset typicality, and algorithmic biases, because the studies used a dataset of a particular disease(s) from a specific location or they used a publicly available dataset. Although the studies present diverse sources of potential bias while developing their proposed DL model using MMDF techniques, each study implemented a particular method to overcome the potential risk of bias. For instance, generative models were used as data augmentation mechanisms to overcome the risk of bias due to data scarcity in [21], while [55] used synthetic data from the generative models to add textual data for multimodal model training. Although these measures helped to overcome the major DL problem (data scarcity) and achieve higher model accuracy, other potential risks of bias could arise as a result of using synthetic data.

Therefore, in every model development project, especially for diagnostic systems, the very first task during the inception of the project should be an in-depth analysis of the available resources (such as data), method(s) to be used, expected output, and potential risk of bias that might arise as a result of using a particular method or resource. Once identified, the potential risk of bias should be evaluated, and properly devised strategies for tackling the potential risk should be in place. Some of the strategies include defining the data requirements (including the type and source of the data); conducting data collection to address scarcity or class imbalance issues; and applying specific strategies for data preprocessing, feature selection, and feature engineering. Additionally, model selection, model optimization, and the use of established evaluation techniques should be considered to ensure the reliability of the models. Finally, professional and ethical criteria are required for any data acquired from external sources, including generative models.

### Ethical Considerations

All the reviewed articles complied with the standard ethical principles, as the studies primarily involved patients and patient data to develop their intended models. However, almost all the previously identified risks of bias can also pose potential ethical issues, since every potential bias has a corresponding problem. Some of the problems include that a DL diagnostic model trained on a relatively small or unbalanced dataset could result in model overfitting, and a model trained on an entirely generated dataset could potentially be challenged when faced with actual real-world data (mostly resulted from data quality differences between the actual and synthetic data). All these flaws result in algorithmic biases, leading to misdiagnosis. Using a misdiagnosing DL model in real-time diagnostic environments is a real danger to the well-being of individuals. It also goes against the ethical principles of the disciplines of medicine and computing. The problem could get much worse if diagnostic systems that use such misdiagnosing models were to be used in underserved areas that have limited resources, because in such areas, the problem of misdiagnosis could result in life-threatening health problems instead of overcoming the burden of diseases. Hence, comprehensive identification and mitigation of all potential sources of bias are required before model deployment. Additionally, as good software engineering practice, a full-fledged testing procedure should be conducted on the diagnostic models to ensure accurate diagnosis.

### Contribution of the Review

Overall, this study’s primary contribution is a critical appraisal of DL-based MMDF techniques for diagnosing skin diseases, including skin NTDs and non-NTD skin diseases. We identified key limitations and the research gaps and proposed the adoption of methods for the diagnosis of skin NTDs. We contributed to the literature by providing, to the best of our knowledge, the first systematic and comprehensive analysis of the applicability of DL-based MMDF techniques for diagnosing both skin NTDs and non-NTD skin diseases. In the process, further research gaps were identified, and corresponding solutions have been forwarded as future research direction. This contributes to the research community by giving insights into the areas that require further investigations, which was the primary goal of this systematic review.

### Conclusion

With the aim of identifying and adopting DL-based MMDF techniques for skin NTD diagnosis, this review analyzed existing DL methods applied to both skin NTDs and non-NTD skin diseases. In this regard, the CNN-based models are the most suitable DL architectures for feature extraction and concatenation, while other DL architectures using RNN are also potential solutions. If text-based features are to be integrated, transformer architectures with attention mechanisms could achieve outperforming results, though it requires further investigation with a particular dataset. Generally, the use of DL-based tools that integrate MMDF techniques has the potential to enhance the diagnosis and management of skin NTDs by improving diagnostic accuracy, especially in resource-constrained settings.

Although studies have demonstrated the use of DL methods using skin images, including 2 recently published studies applying MMDF, there is a notable research gap regarding the implementation of MMDF techniques for the diagnosis of skin NTDs. This systematic review, thus, identified 3 critical gaps and suggested potential solutions for future work. First, due to the lack of previous studies applying MMDF techniques for the diagnosis of skin NTDs, this review has extended its scope to include studies focusing on non-NTD skin diseases, representing 86% of the total analyzed articles. In this regard, the findings of this review highlight that further studies are required regarding the application of DL-based MMDF techniques for the diagnosis of skin NTDs. Second, the availability of high-quality datasets (having diversified modalities and proper annotation and of sufficient quantity to train DL model) remains a challenge for conducting DL-based studies for skin NTD diagnosis. This scarcity hinders potential studies in the area, which highlights the need for efforts to develop and share large-scale, quality datasets. In addition, further investigations are required to research the applicability of synthetic data generated using generative models to address data scarcity and maintain dataset quality when developing diagnostic models for skin NTDs. Finally, the selected studies underscored promising results toward the application of MMDF techniques for skin NTDs with limited modalities of patient data (image and metadata). However, further studies are required to demonstrate the applicability of MMDF techniques for skin NTDs using more than 2 modalities, such as the combination of skin images, structured (tabular) patient data, and textual descriptions.

## Appendix

Multimedia Appendix 1 Article sources, list of search terms used to search for articles, and overall analysis of articles used for the final analysis of this systematic review.

Multimedia Appendix 2 PRISMA checklist.

## Abbreviations

AI: artificial intelligence
AJOL: African Journals Online
BERT: bidirectional encoder representations from transformers
BiGRU: bidirectional gated recurrent unit
BiLSTM: bidirectional long short-term memory
CL: cutaneous leishmaniasis
CNN: convolutional neural network
DCNN: deep convolutional neural network
DL: deep learning
DNN: deep neural network
GAN: generative adversarial network
LLM: large language model
LR: logistic regression
LSTM: long short-term memory
ML: machine learning
MLP: multilayer perceptron
MMDF: multimodal data fusion
PNAS: progressive neural architecture search
PRISMA: Preferred Reporting Items for Systematic Reviews and Meta-Analyses
RF: random forest
RNN: recurrent neural network
SENet: Squeeze-and-Excitation Network
STH: soil-transmitted helminthiases
SVM: support vector machine
ViT: vision transformer
WHO: World Health Organization
XGB: eXtreme gradient boost
