# Supplementary material for: Application of Deep Learning-Based Multimodal Data Fusion for the Diagnosis of Skin Neglected Tropical Diseases: Systematic Review
Source: JMIR AI. 2025 Dec 4;4:e67584. doi: 10.2196/67584 (PMC12715462; doi:10.2196/67584)
Supplement: Multimedia Appendix 1 [file ai_v4i1e67584_app1.doc]

Multimedia Appendix I

Article sources and the List of search terms used to search for articles

**Table S1: Summary of the article sources and the search terms used for the data sources**

| **No.** | **Source Type** | **Article Source** | **Search Terms Used** | **Search Terms applied** |
| --- | --- | --- | --- | --- |
| **1** | Search Engine | Google Scholar | 1. [((((("Neglected Tropical Diseases") OR ("NTDs") OR ("skin related Neglected Tropical Diseases)" OR (“Skin-Related Neglected Tropical Diseases”) OR ("Skin NTDs") OR (“tropical diseases”)) AND (("Diagnosis") OR ("diagnostic model") OR ("classification model"))) AND (("Deep Learning") OR ("DL") or ("Convolutional Neural Network") OR ("CNN") OR ("Deep Neural Network") OR ("DNN") OR ("Recurrent Neural Network") OR ("RNN"))) AND (("Data Fusion") OR ("Data Fusion Techniques") OR ("Data Fusion methods") OR ("Multimodal medical Data") OR ("Multimodal Data Fusion") OR ("Multimodal Data Fusion Techniques")))], 2. [((((“Machine Learning Methods”) OR (“ML Methods”) OR (“Deep Learning”) OR (“DL”)) AND ((diagnostic model) OR (diagnostic system) OR (diagnostic tool))) AND (((“skin diseases”) AND (“skin images”)) AND (medical record))) AND ((data fusion) OR (“multimodal data fusion”))))], 3. [((("Neglected Tropical Diseases") OR ("NTDs") OR ("skin related Neglected Tropical Diseases)" OR (“Skin-Related Neglected Tropical Diseases”) OR ("Skin NTDs") OR (“tropical diseases”)) AND ((“Artificial Intelligence”) OR (“AI”) OR (“machine learning”) OR (“ML”) OR ("Deep Learning") OR ("DL") OR ("Convolutional Neural Network") OR ("CNN") OR ("Deep Neural Network") OR ("DNN") OR ("Recurrent Neural Network") OR ("RNN")) AND (("Diagnosis") OR ("NTDs Diagnosis") OR ("diagnostic model") OR ("Diagnostic System") OR ("Diagnostic Tool")))], 4. [(((("Skin Neglected Tropical Diseases") OR ("Skin NTDs") OR (“skin diseases”) OR (“non-NTD skin diseases”)) AND (("Diagnosis") OR ("diagnostic model") OR (“skin diseases classification”) OR (“skin images classification”))) AND ((("Deep Learning") OR ("DL") or ("Convolutional Neural Network") OR ("CNN") OR ("Deep Neural Network") OR ("DNN") OR ("Recurrent Neural Network") OR ("RNN")) AND (("Data Fusion") OR ("Multimodal medical Data") OR ("Multimodal Data Fusion") OR ("Multimodal medical Data Fusion") OR ("Multimodal Skin Data Fusion"))))], 5. [(((deep learning) AND ((diagnostic model) OR (diagnostic system) OR (diagnostic tool)) AND (skin diseases) AND (skin images)) AND (medical record)) AND (data fusion))], and | **Search terms**  **1 – 5**  **are All Applied On All Sources (Except for Non-Database Sources)** |
| 2 | Article Database | IEEE Xplore, ScienceDirect, PubMed, AJOL, Scopus, and Web of Science |
| **3** | Publisher | Springer, MDPI, PLOS NTD, and Tropical Medicine and Health |
| **4** | Specialized Journals (Targeted Search) | AJOL, Tropical Medicine and Health, PLOS NTD, |
|  | Article Database | IEEE Xplore | 1. [("All Metadata":analysis) AND ("All Metadata":Deep Learning techniques) OR ("All Metadata":deep learning methods) AND ("All Metadata":skin disease diagnosis) AND ("All Metadata":multimodal medical data fusion)] has been used to search articles specifically from the IEEE Explore database. | Applied on IEEE Explore |
| **5** | Gray Article Source ( Gray Literature) | Non-Database Sources: all online sources that are not necessarily indexed | - All possible combinations of the keywords, except the search terms identified here, are used to search for data - External Sources, such as academic social networks using Mendeley, organizational web portals (such as WHO), academic institutions (such as university web portals), and related sourced were consulted, | No specific search terms were used |

Overall analysis of articles used for the final analysis of this systematic review

**Table S2: Review of the DL-based multimodal data fusion techniques for the diagnosis of skin diseases**

| **Ref.** | **Pub. Year** | **Study Method / Approach Used** | | **Disease(s) Selected** | **Dataset(s) Used** | **Algorithm(s) Used** | **Performance / Accuracy Results Achieved** | **Recommendations Forwarded** |
| --- | --- | --- | --- | --- | --- | --- | --- | --- |
| [8] | 2022 | collecting skin lesion images along with clinical & demographic data, model training, including cross-validation and validation), employ CNN, analyze images, using ML algorithms (elastic-net logistic regression, XGB, and RF) to combine image analysis with metadata | | Leprosy | dataset collected at a leprosy clinic in Brazil (1229 skin lesion images and 585 sets of metadata from 222 patients diagnosed with leprosy) | XGB, RF, LR, and 3 different models used (Model 1 for image analysis using Inception-v4, ResNet-50, Model 2- handling metadata, & Model 3 for integrating Model 1 with Model 2 using elastic-net LR | Model1-moderate (best accuracy of 66.6% & AUC of 74.56%, elastic-net LR on metadata (Model2- higher accuracy (90%) and AUC of 96.46%), RF on patient information achieved the highest AUC of 98.74% on the testing patients | further validation with larger, more diverse datasets, including images captured via smartphone apps in real-world settings, training the model on various skin types and in different geographical locations, |
| [21] | 2025 | GANs-based Multimodal Diagnostic Framework for the detection of Malaria and Dengue (using the GANs for data augmentation and an ensemble of Bidirectional BiLSTM, Bidirectional Gated Recurrent Unit (BiGRU), & RNN models for classification), and includes an experimental evaluation | | Malaria and Dengue Detection | a set of clinical samples (Malaria and Dengue cases), and specific sources Dengue Surveillance Dataset (DSD), ImageNet Medical Images Dataset (INMIM), GHO | GANs, ensemble of BiLSTM, BiGRU, & RNN, checking the performance of this framework (GMBLGMRD) against DHL ELM CNN, DFM (Deep Forest Methods), CRNN (Convolutional-RNNs) | Precision - 96.06%, consistently higher (accuracy-91.96%, & AUC 88.32%), higher recall (94.98%), all at 665k NTS, improved performance (precision by 4.9%, accuracy by 3.5%, recall by 3.5%, and AUC by 4.5%), | demonstration of the potential for the other infectious diseases (NTDs), demonstration of other aspects (integrating multi-omics data, real-time monitoring, enhancing model adaptability, add explainability) |
| Articles that Implemented MMDF Methods for Non-NTD Skin Diseases | | | | | | | | |
| [9] | 2020 | Multiplication-based DF, using the metadata | No specific disease selected | | ISIC’2018 and three types of metadata | CNN, the color constancy algorithm | outperforms traditional baseline approaches (p-values are smaller than 0.05) | Exploring effects of more types of metadata for more skin diseases |
| [10] | 2021 | Combining images and metadata features: the (MetaBlock) | Skin Cancer | | ISIC 2019 and PAD-UFES-20 | CNN: using 5 pre-rained models | Performs better than the other combination approaches in 6 out of 10 scenarios. |  |
| [11] | 2021 | Performance analysis of classifiers, and a naive combination of patient data and an image classifier | Skin Cancer | | Collected 431 WSIs from two different Labs with patient information | CNN | CNN: AUROC-92.30% ± 0.23% & balanced accuracy of 83.17% ±0.38%), naive strategy: accuracy to 86.72% ±0.36%. | Pointed that the results achieved need to be confirmed systematically in larger studies with diverse data sets. |
| [12] | 2022 | A DNN-based multi-modal classifier using wound images and their locations:- body map development, multi-modal network | Wound Diagnosis | | developed (AZH DS), public DS (Medetec DS), and developed a mixed DS (AZHMT DS) | classificaion NNs (AlexNet + MLP, AlexNet + LSTM, ResNet50 + MLP, VGG16 + LSTM) | Max. Acc. on mixed class: varies from 82.48 to 100% the max. acc. on wound-class varies from 72.95 to 97.12% in various experiments | Adding more modalities and more data, and using more specific wound image classifier and wound location classifier networks |
| [48] | 2018 | Combining multiple imaging modalities (dermatoscopic & macroscopic) with patient metadata | 5 cases (such as melanoma) | | New DS composed of 2917 cases, from five classes (five selected diseases) | CNN , Random Forrest classifier, 2 ResNet-50 architectures | binary melanoma detection (AUC 0.866 vs 0.784) and in multiclass classification (mAP 0.729 vs 0.598) | Integrating more benign non-excised skin and further stratify them based on suspicion |
| [49] | 2022 | a DNN with two encoders and application of a multimodal fusion module with intra-modality self-attention and inter-modality cross-attention | Skin Cancer | | PAD-UPES-20 | CNN: CNN models (ResNet-50) | ACC (0.768 ± 0.022) and BACC (0.775 ± 0.022) and claimed outperforming other metadata fusion methods (MetaNet (P = 0.035) and MetaBlock (P = 0.028)) | Integrating the model into smartphone as a potential and handy tool to screen for skin disease and skin cancer |
| [50] | 2022 | Multimodal Transformer: Vision Transformer (ViT) model, Soft Label Encoder (SLE), and a Mutual Attention (MA) block | Skin disease | | A private DS collected (760 images) & benchmark DS of the ISIC 2018 | CNN: CNN models (ResNet101, Densenet121) and ViT models | Private DS (accuracy: 0.816, which is better than other popular networks) & On ISIC 2018 DS (accuracy: 0.9381 and an AUC of 0.99) | To focus on image feature extraction and also, the data collection and data cleaning of skin diseases |
| [51] | 2022 | Medical image analysis: Preprocessing, feature extraction, and classification/diagnosis, & the hold-out technique to split the dataset | Actinic Keratosis, KBL, NV, BCC, DF, MEL and VASC | | HAM10000 dataset | CNN: 6 CNN pre-trained models, Hyper-Parameter Optimization (HPO) algorithms | Av. acc, sensitivity, specificity, precision, & disc similarity coefficient (DSC) of around 99.94%, 91.48%, 98.82%, 97.01%, and 94.00%, respectively | Testing other DL techniques to improve the classification accuracy and using other benchmark datasets with different skin disorders |
| [52] | 2023 | Mapping heterogeneous data features, fusion of clinical skin image & patient clinical data, feature extraction & attention mechanisms | Skin Cancer | | PAD-UFES-20:- skin images and patient information | CNN: networks (VGGNet19, ResNet50, DenseNet121 & Inception-V3) | Achieved accuracy of 80.42% (an improvement of about 9% compared with the model accuracy using only medical images) | expanding scope of data collection: various types of imaging data (CT, MRI, US) and their corresponding clinical information |
| [53] | 2023 | TFormer: use of transformer as the feature extraction backbone (with dual-branch HMT), use of a MTP block to integrate features from image modalities and patient meta-data, and use of "divide and conquer" strategy (first fusing image modalities and then aggregating with meta-data) | Skin Lesion | | Derm7pt dataset that contains multimodal data (dermoscopic images, clinical images, and patient meta-data with eight types of labels) | Swin Transformer (Image feature extraction), Dual-branch HMT blocks with WMCA (for Image modality fusion), MLP (Meta-data feature extraction), MTP block (Fusion of image features and meta-data) | average accuracy of 77.99% and a diagnostic accuracy of 80.03% on the Derm7pt dataset, also showed superiority in terms of sensitivity, specificity, precision, and F1-score across most labels compared to other methods | leveraging transformer structures for feature extraction and the designed HMT and MTP blocks facilitate better integration of multi-modal data |
| [54] | 2025 | prediction of skin cancer (clinical metadata & dermoscopic images), TL (EfficientNetB3 for extraction of from dermoscopic images, and TabNet for processing the clinical metadata | Skin Cancer | | benchmark datasets (ISIC 2018, ISIC 2019, and HAM10000) | EfficientNetB3 (for Image feature extraction), TabNet (for Clinical metadata processing), and Attention-based fusion mechanism for fusion | Consistently higher accuracy 98.69% in classifying skin cancer on multiple dataset like ISIC 2018/19 and HAM10000 datasets with less variation | application in other medical fields requiring multimodal data integration, extending the research by integrating genetic data for a better understanding of skin ailments |
| [55] | 2025 | using two distinct LLMs (GPT-4-turbo and Gemini-Pro-1.5) for generating clinical notes for each patient’s skin cancer images, using cross-evaluation & consensus scoring methods (using metrics of BLEU score, ROUGE score, Overlap Coefficient, and Jaccard index), | Skin Cancer | | the SIIM-ISIC Melanoma Classification dataset (Kaggle) | Baseline model (for image-only) - ResNet-50, Multimodal models - BERT-ResNet and ALBEF, LLMs (for synthetic clinical note generation - OpenAI’s GPT-4-turbo and Google’s Gemini Pro) | ALBEF model: highest (accuracy-99.51%, precision 96.13%, recall of 97.95%, and F1-score of 97.03%), GPT-4-turbo outperformed Google Gemini Pro 1.5 in generating detailed and contextually rich clinical notes, with higher BLEU & ROUGE scores (0.87 & 0.84,compared to 0.81 & 0.78 for Gemini Pro 1.5) | addressing ethical implications (particularly regarding data privacy and the potential biases inherent in synthetic data) |
